# Rare overlap of subtype and phenotype: HER2-positive pleomorphic invasive lobular carcinoma presenting with an inflammatory breast cancer phenotype: a case report and targeted treatment strategy

**DOI:** 10.3389/fonc.2026.1810319

**Published:** 2026-04-13

**Authors:** Zhifeng Xiong, Gendou Zhou, Na Zhou, Luyao Wang, Gang Liu, Qiong Zhang, Gang Lyu

**Affiliations:** 1 Department of Breast, Chongqing Hospital of Traditional Chinese Medicine, Chongqing, China; 2 Medical Oncology Department, Chongqing University Fuling Hospital, Chongqing, China; 3 College of Nursing, Chongqing Medical and Pharmaceutical College, Chongqing, China; 4 Department of Breast, The Third Affiliated Hospital of Beijing University of Chinese Medicine, Beijing, China

**Keywords:** HER2-positive breast cancer, inflammatory breast cancer, pleomorphic invasive lobular carcinoma, pyrotinib, trastuzumab deruxtecan

## Abstract

Pleomorphic invasive lobular carcinoma (PLC) is a rare and aggressive subtype of invasive lobular carcinoma. When it presents with an inflammatory breast cancer (IBC) phenotype, due to its rarity, this can lead to substantial diagnostic and therapeutic challenges. Herein, we report the case of a 53-year-old woman presenting with a rapidly enlarging left breast mass, diffuse edema, erythema, and pain; these symptoms have affected the entire breast over the course of one month. Imaging revealed diffuse parenchymal abnormalities and nodal involvement, while biopsy indicated PLC, histologic grade III, with HER2 amplification and complete absence of hormone receptors. Clinical and radiological assessment demonstrated nodal involvement (supraclavicular, cervical, and bilateral axillary) and distant osseous metastases, consistent with stage cT4N3M1 disease. The patient received first-line docetaxel, trastuzumab, and pyrotinib (PyroHT) and achieved a partial response; however, progression was observed after ten cycles. Following multidisciplinary review, her treatment was changed to trastuzumab deruxtecan (T-DXd), resulting in significant symptom relief and radiologic regression of the breast lesions. This case highlights the importance of early biopsy in rapidly progressive breast disease and that treatment for rare histologic subtypes, such as PLC, should be guided by molecular profiling rather than histology alone. Sequential HER2-targeted therapy provided meaningful clinical benefit, though resistance ultimately developed. We present the case of a 53-year-old woman with HER2-positive advanced PLC presenting with an IBC phenotype, providing insight into the diagnostic process and treatment course in this rare clinical scenario.

## 1 Introduction

Invasive lobular carcinoma (ILC) is the second most common histological subtype of breast cancer, accounting for approximately 10–15% of all invasive cases (1). Among its variants, pleomorphic ILC (PLC) represents a particularly rare and aggressive form, comprising only about 5–15% of ILC cases and less than 1% of all breast cancers (2). PLC shares several key biological hallmarks with classic ILC (C-ILC), including a diffuse infiltrative growth pattern and loss of E-cadherin expression (3); however, it can be distinguished by marked nuclear atypia, higher histologic grade (4). At the molecular level, PLC also exhibits a genetic landscape that differs from C-ILC, such as recurrent alterations involving TP53, HER2 and PIK3CA, which are associated with increased proliferative capacity and invasive behavior, and are often linked to poorer outcomes. However, when PLC presents with features of inflammatory breast cancer (IBC), it creates a rare and diagnostically challenging situation that necessitates early recognition and intensive multimodal treatment (5).

Although the incidence of IBC is relatively low, the disease progresses rapidly and is recognized as one of the most aggressive forms of breast cancer. Approximately one-third of patients present with distant metastases at diagnosis (6), and due to its poor prognosis, it accounts for up to 10% of breast cancer-related deaths (6). Its hallmark is the rapid onset of skin changes such as erythema, edema, warmth, and breast enlargement, which can resemble infectious mastitis and contribute to diagnostic delays (7). The diagnosis is based mainly on clinical findings, with histopathology serving only a supportive role; while features such as dermal lymphatic invasion, diffuse skin thickening, and absence of a distinct mass are often observed, their inconsistency makes both clinical and radiologic assessment particularly challenging (8).

The coexistence of PLC with an IBC phenotype adds considerable diagnostic and therapeutic challenges. ILC is characterized by minor radiologic findings and a high frequency of multifocal or multicentric disease, which complicates accurate staging and surgical planning (3). When these features are accompanied by the diffuse edema, erythema, and skin thickening typical of IBC, interpretation becomes even more complex, often obscuring lesion boundaries and delaying recognition. The pleomorphic variant adds another layer of difficulty because of its higher grade, greater proliferative activity, and association with worse clinical outcomes compared with classic ILC (9). Thus, these overlapping features create a clinical scenario in which precise diagnosis requires careful correlation of imaging and histopathology, while effective treatment demands an aggressive multimodal approach.

Human epidermal growth factor receptor 2 (HER2) positivity in breast cancer, occurring in approximately 15-20% of cases, has improved treatment options through the development of targeted therapies (10). The introduction of trastuzumab and subsequently trastuzumab deruxtecan (T-DXd), an antibody-drug conjugate, has significantly improved outcomes for patients with HER2-positive metastatic breast cancer, offering new hope for patients with advanced disease (11). T-DXd has also demonstrated remarkable efficacy in heavily pre-treated HER2-positive metastatic breast cancer, including patients with brain metastases (12). Several small-sample retrospective studies have previously explored the clinicopathological features of IBC with ILC characteristics (13, 14), but none have analyzed the specific subtypes of ILC, nor have they elaborated in detail on the diagnosis, treatment, follow-up protocols and prognosis-related conditions. To date, limited data are available regarding the efficacy of docetaxel, trastuzumab, and pyrotinib (PyroHT) and T-DXd in patients with HER2-positive advanced PLC presenting with an IBC phenotype.

PLC with an IBC phenotype is rare, and current evidence offers little guidance on optimal management. The lack of standardized strategies underscores the value of individual case reports in elucidating diagnostic challenges, informing therapeutic decision-making, and assessing clinical outcomes.

## 2 Case presentation

A 53-year-old Chinese woman presented on 31 March 2024, with a three-month history of a rapidly enlarging left breast mass. Initially, self-examination revealed a patch-like lesion measuring approximately 3 × 2 cm in the upper outer quadrant of the left breast, but within one month, the lesion, along with diffuse cutaneous edema and erythema,had expanded to involve the entire breast and was associated with swelling, marked pain, and a mild increase in local temperature. The patient initially sought care at her local hospital, where clinicians recommended a core-needle biopsy to obtain a definitive diagnosis, which she declined for personal reasons and subsequently received a five-day course of intravenous antibiotics at a local clinic. However, this treatment did not result in improvement of the breast mass or cutaneous symptoms, and she did not return for additional medical interventions until her later presentation at our institution.

**Figure 1:**
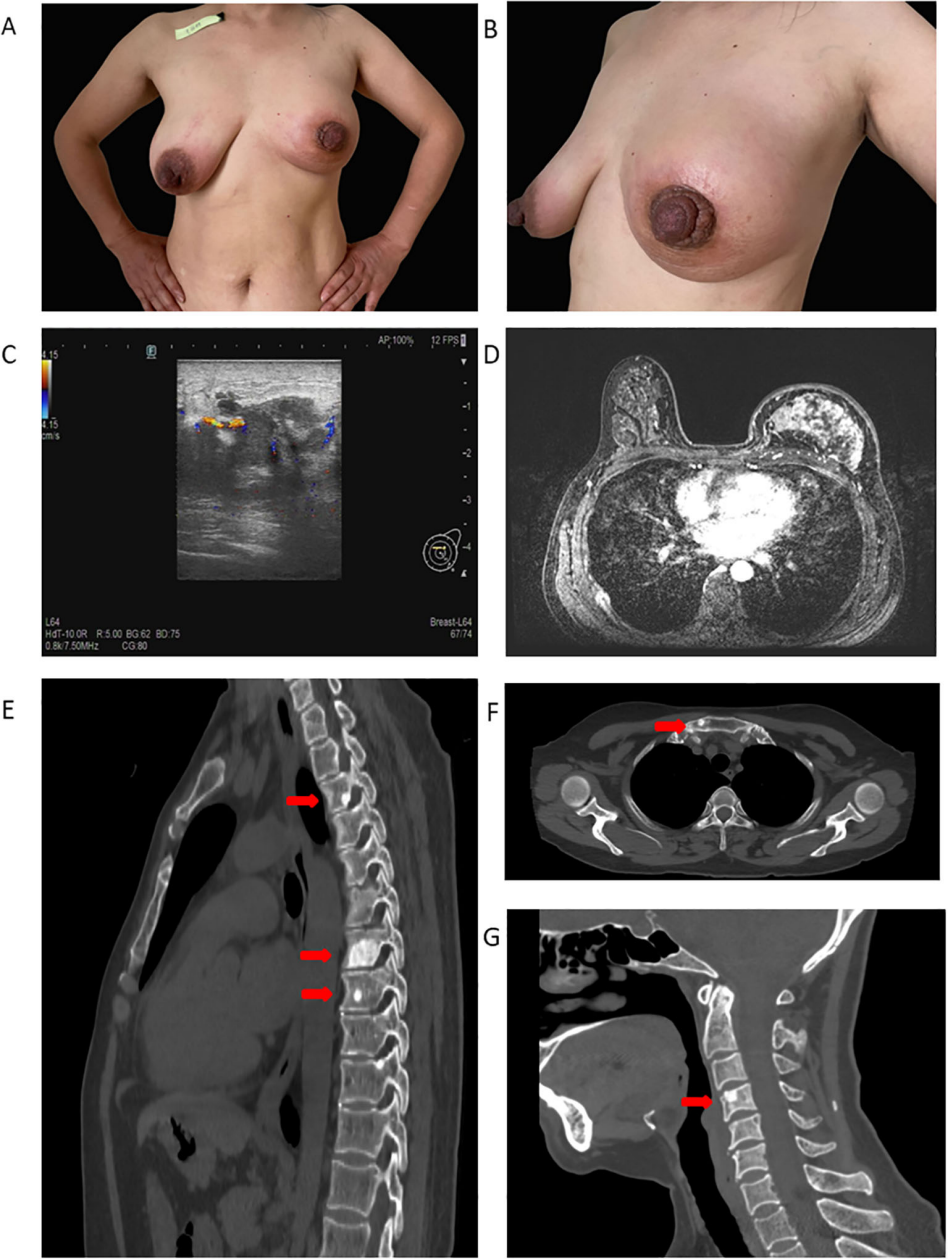
Imaging features at initial presentation. **(A, B)** Frontal and lateral photographs of the breast at the initial visit. **(C)** Color Doppler ultrasonography demonstrating diffuse hypoechoic and heterogeneous parenchymal echoes involving nearly the entire left breast. **(D)** Breast MRI showing diffuse enhancement, skin thickening, and an ill-defined nodular lesion with a type III washout kinetic curve. **(E–G)** Contrast-enhanced CT showing focal osteolytic destruction involving parts of the thoracic vertebrae, cervical vertebrae, and the manubrium.

Upon re-examination, there was noticeable asymmetry between the breasts. The left side showed diffuse cutaneous edema and erythema with increased local temperature and marked tenderness (**Figures 1A, B**). A firm, poorly mobile mass measuring approximately 14 × 14 cm occupied nearly the entire breast. The nipple-areolar complex was fixed to the underlying tissue, although no nipple discharge was detected. Bilateral axillary lymphadenopathy was present, with nodes measuring approximately 2 × 1.5 cm. In addition, the left supraclavicular fossa appeared full, and enlarged lymph nodes were palpable in this region.

Color Doppler ultrasound of the breast (**Figure 1C**) demonstrated diffusely decreased and heterogeneous parenchymal echogenicity throughout the left breast. Color Doppler imaging revealed disorganized strip-like vascular signals, and pulsed-wave Doppler (PW-Doppler) detected an arterial waveform with a resistive index of 0.82. Based on these findings, the lesion was categorized as Breast Imaging Reporting and Data System (BI-RADS) 4C. Bilateral axillary lymph nodes were enlarged, more prominent on the left, raising suspicion for metastatic involvement. Mammography was not performed because severe pain prevented adequate breast compression. Targeted lymph-node US identified multiple abnormal nodes in the left neck, supraclavicular region, and bilateral axillae, characterized by cortical thickening and mixed Doppler signals. No suspicious nodes were detected in the right neck, right supraclavicular region, or bilateral inguinal regions.

Contrast-enhanced breast MRI (**Figure 1D**) showed that both breasts were predominantly composed of glandular tissue. In the left breast, several nodular and irregular patchy lesions were observed, appearing hypointense on T1-weighted images and iso- to hypointense on T2-weighted images, with mildly increased signal intensity on diffusion-weighted imaging. After administration of contrast agent, the lesions exhibited marked heterogeneous enhancement with a type III washout curve, a kinetic pattern typically seen in malignant breast tumors. The overlying skin was diffusely thickened, and the margin between the lesion and the left nipple was poorly defined. Soft tissue swelling of the left chest wall was also present, and within the scanned area, multiple enlarged lymph nodes were identified in both axillae. Contrast-enhanced chest CT further confirmed multiple enhancing soft-tissue masses in the left breast with associated skin thickening. Overall, these findings suggested metastatic involvement of the bilateral axillary and internal mammary lymph nodes, with possible bone metastases involving the thoracic spine (**Figure 1E**), the manubrium (**Figure 1F**), and the C4 vertebral body (**Figure 1G**).

**Figure 2:**
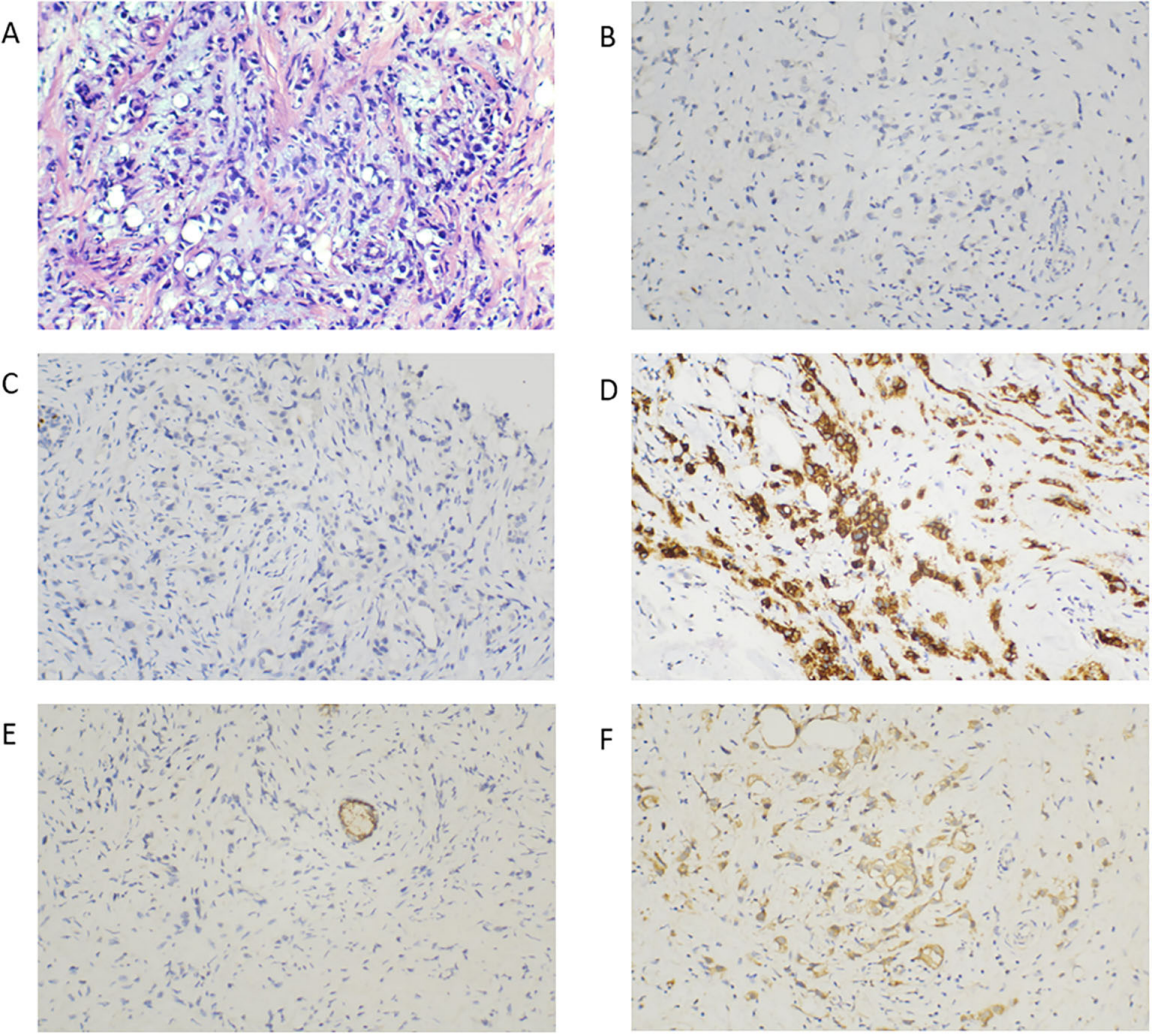
Representative histopathological findings of the primary left breast lesion. **(A)** Hematoxylin and eosin staining showing tumor cells infiltrating the stroma as small nests, single cells, and single-file linear arrays; nuclei are pleomorphic with conspicuous nucleoli, eccentric nuclear positioning is noted, mitotic figures are present, and abundant intracytoplasmic vacuoles impart a signet-ring–like appearance (H&E, ×100). **(B–F)** Immunohistochemistry of the primary lesion showing negative ER **(B)** and PR **(C)**, HER2 (3+) **(D)**, loss of E-cadherin expression **(E)**, and positive p120 catenin staining **(F)** (×100).

**Figure 3:**
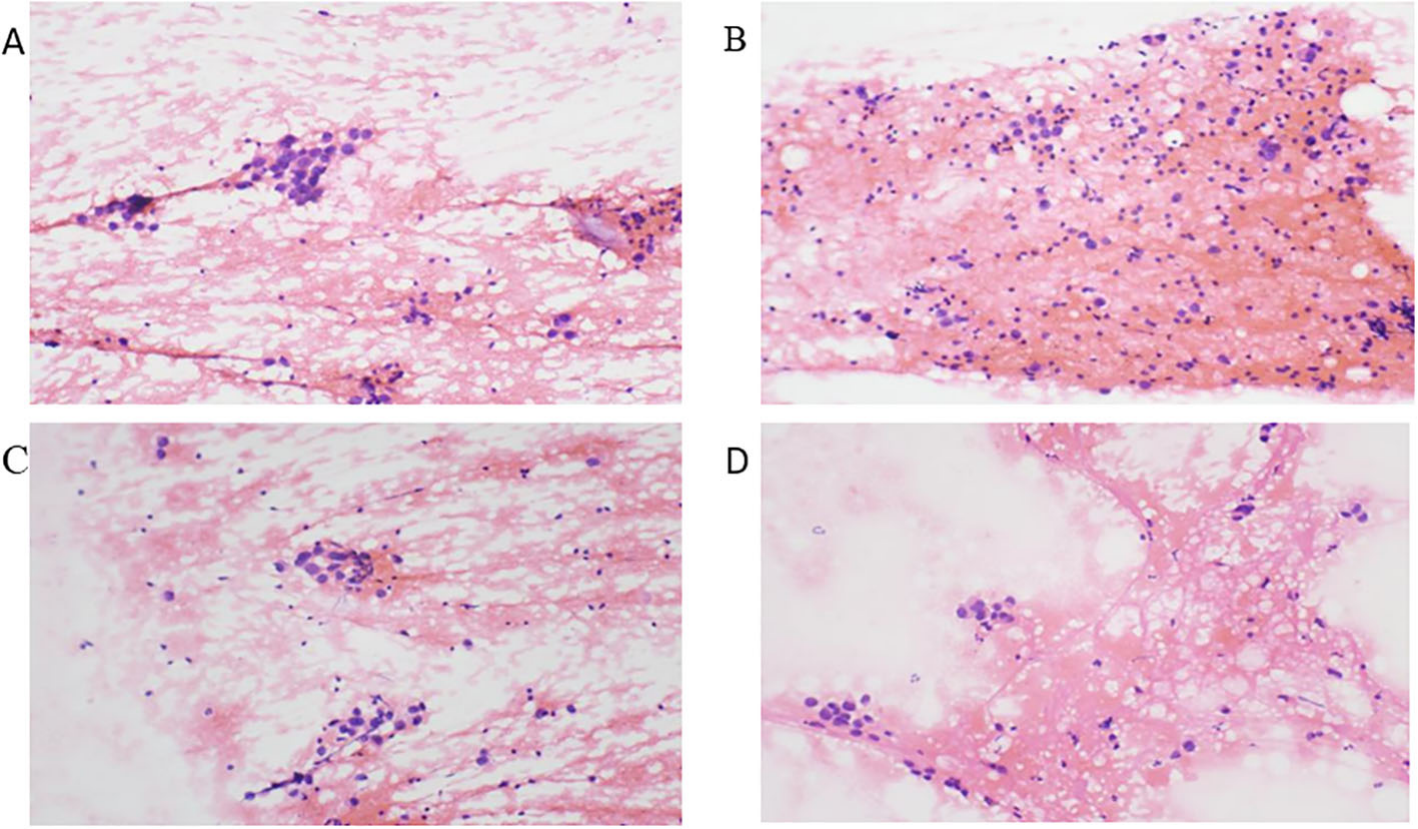
Histopathological analysis of lymph node biopsies. Biopsies taken from the left axillary lymph node **(A)**, right axillary lymph node **(B)**, left supraclavicular lymph node **(C)**, and left level IV cervical lymph node **(D)** showing malignant cells of suspected epithelial origin (×400).

A core needle biopsy of the left breast lesion revealed invasive carcinoma with morphological features consistent with pleomorphic invasive lobular carcinoma, histologic grade III. Immunohistochemistry showed HER2 positivity (3+), while negative for estrogen receptor (ER) and progesterone receptor (PR). The Ki-67 labeling index was ~40%, indicating high proliferative activity. Cytoplasmic staining for p120 and TRPS1 was positive, supporting lobular differentiation, while markers including MHC, β-catenin, E-cadherin, CK5/6, calponin, and p63 were negative (**Figure 2**). Fine-needle aspiration of the left supraclavicular, left cervical, and bilateral axillary lymph nodes revealed the presence of malignant adenocarcinoma cells (**Figure 3**). According to the American Joint Committee on Cancer (8th edition) (15), diagnostic criteria for IBC (T4d) are based on clinical diagnosis by a rapid onset of diffuse erythema and edema (or peau d’orange) involving approximately at least one-third of breast skin, with or without an underlying palpable mass, the onset of symptoms in IBC should be rapid, within no more than 6 months. This patient presented with a rapidly enlarging left breast mass, diffuse edema, erythema, and pain, and these symptoms involved the entire left breast within one month, consistent with the above clinical diagnostic criteria for IBC. Skin biopsy was recommended to confirm histologically whether dermal lymphatic invasion was present, but the patient declined. Taken together, the patient was diagnosed with HER2-positive, hormone receptor-negative PLC of the left breast presenting with an IBC phenotype. The disease was staged as cT4N3M1 (stage IV), with metastases to the left supraclavicular, left cervical, and bilateral axillary lymph nodes, as well as to the thoracic vertebrae, manubrium, and C4 vertebral body.

After multidisciplinary tumor (MDT) board discussion and comprehensive assessment according to the Chinese Society of Clinical Oncology guidelines, On 19 April 2024 systemic therapy was initiated with the PyroHT, administered in 21-day cycles. The regimen consisted of docetaxel 75 mg/m² × 1.4 m^2^ (105 mg), trastuzumab with a loading dose of 8 mg/kg × 48 kg (380 mg) followed by maintenance doses of 6 mg/kg × 48 kg (300 mg), and oral pyrotinib maleate 400 mg once daily. Zoledronic acid 4 mg was given intravenously every 28 days as anti-resorptive therapy for bone metastases.

Tumor responses were evaluated according to the Response Evaluation Criteria in Solid Tumors (RECIST) version 1.1. Local tumor burden and locoregional disease extent were serially assessed using breast color Doppler ultrasound, contrast−enhanced breast MRI, and clinical examination. Systemic staging and evaluation for distant metastases were performed using contrast−enhanced chest CT and breast MRI. PET−CT was not performed on this patient due to financial constraints. Bone metastases were diagnosed radiologically based on typical features on contrast−enhanced CT and MRI. Biopsy of the bone metastases was not performed because the core needle biopsy of the primary breast lesion confirmed the histologic subtype (pleomorphic invasive lobular carcinoma) and definitive biomarker status (HER2−positive, ER−negative, PR−negative), and the patient declined the procedure.

**Figure 4:**
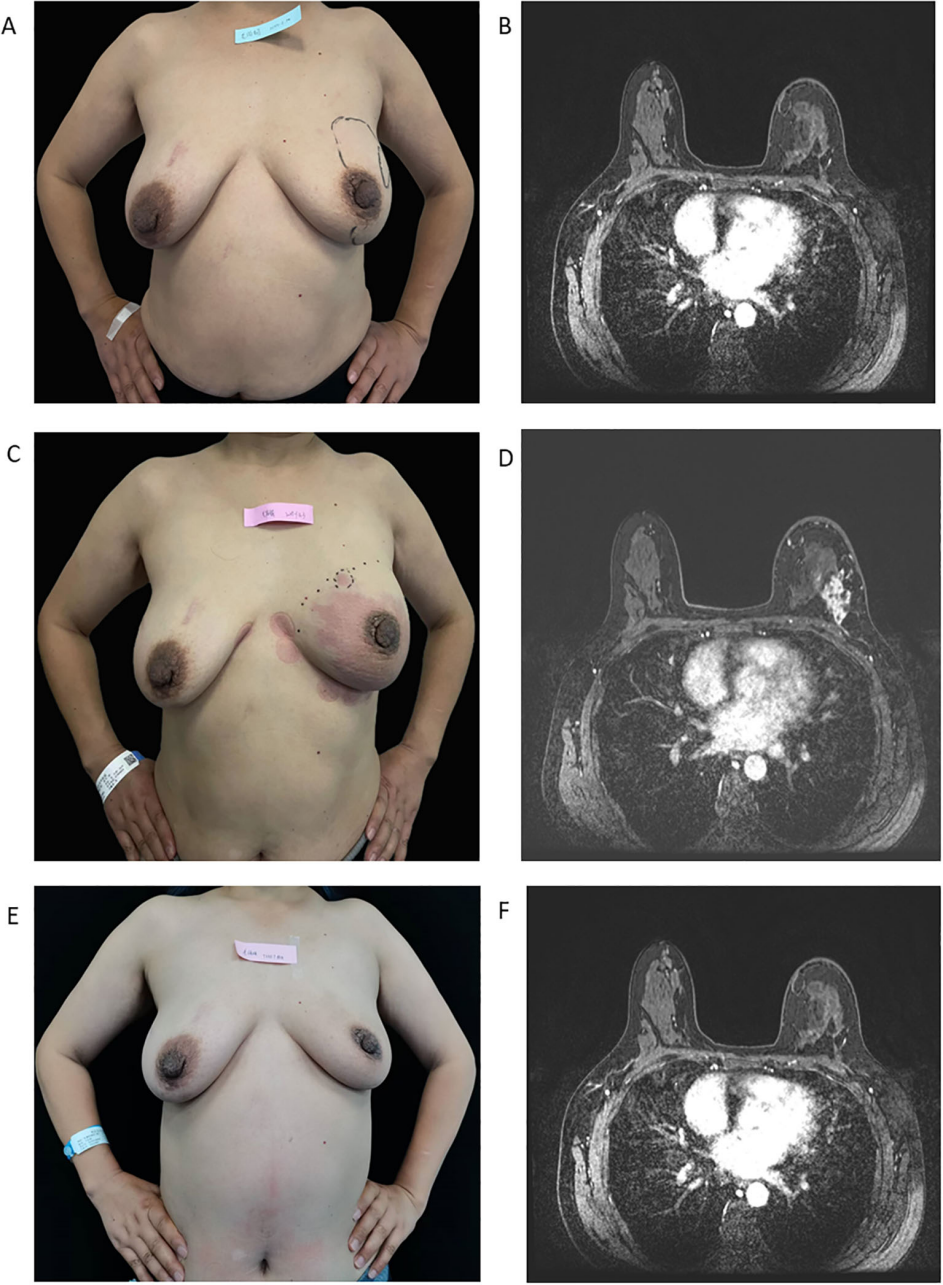
Treatment response assessment by serial breast photographs and MRI. **(A, B)** Breast photographs and MRI after four cycles of docetaxel plus trastuzumab plus pyrotinib, showing resolution of left breast erythema, improvement in edema, and reduction in the extent of the lesions. **(C, D)** Breast photographs and MRI after ten cycles of docetaxel plus trastuzumab plus pyrotinib, showing recurrence of erythema and increased lesion extent compared with the prior assessment, consistent with disease progression. **(E, F)** Breast photographs and MRI after four cycles of T-DXd, showing improvement of erythema and edema with a reduced extent of the lesions.

After four cycles, on 19 July 2024, clinical examination (**Figure 4A**) and breast MRI (**Figure 4B**) showed regression of the left breast lesions, and the overall response was assessed as a partial response. During this period, no severe toxicities such as diarrhea or hepatic injury occurred, and the patient’s body weight increased to 52 kg, indicating an improvement in general condition. Based on these findings, the regimen was continued with minor dose adjustments, and the progression-free survival (PFS) was 7 months. However, after the tenth cycle, On 15 December 2024, the patient developed progressive disease, presenting with rebound enlargement of the breast mass accompanied; erythema, edema and peau d’orange recurred within 1 week involving more than one-third of the left breast (**Figure 4C**). MRI (**Figure 4D**) confirmed a marked increase in tumor burden compared with the October 2024 evaluation. A second MDT board review was convened, and in accordance with current treatment recommendations, the therapeutic strategy was changed on 2 January 2025 to T-DXd (DS-8201), administered every 21 days at a dose of 5.4 mg/kg × 52 kg (280 mg). After four cycles of T-DXd, the breast mass decreased in size, and the erythema and pain improved (**Figures 4E, F**). Thereafter, T-DXd was continued in combination with zoledronic acid as rescue therapy until further progression. To date, the PFS has reached 13 months.

## 3 Discussion

This case illustrates the aggressive clinical course and therapeutic complexity of PLC presenting with an IBC phenotype, a rare combination of two uncommon diseases. Importantly, PLC is a histologic subtype, whereas IBC is a clinical phenotype, and in practice, this combination may be easily missed because the dominant presentation is often “inflammatory”, that is diffuse skin redness, swelling, warmth, and pain can resemble mastitis, When these features overlap, the condition is easily mistaken for mastitis, as in our case, whereby our patient thought it was an inflammation and self-administered intravenous antibiotics, which resulted in not significant observed benefit. Limited recognition of this rare presentation (“atypical breast lesion with skin redness and swelling”) in primary-care settings, together with inadequate patient awareness, can contribute to delayed diagnosis. Once the diagnosis is postponed, the disease may progress rapidly with early systemic dissemination, leading to a sharp worsening of prognosis (7).

The overlap of PLC and IBC is not an incidental clinical combination, but rather reflects profound biological commonality and synergistic effects. At the molecular phenotypic level, Both exhibit high aggressiveness, heterogeneity, and metastatic potential: PLC is defined by E-cadherin loss, discohesive cell growth, and cytoplasmic p120 positivity (promoting diffuse infiltration and vascular invasion), while IBC features dermal lymphatic tumor emboli, extensive skin involvement, and rapid systemic spread. Their consistent biological behavior drives early dissemination, rapid progression, and therapeutic resistance, forming a vicious cycle (high aggressiveness → rapid progression → delayed recognition) that challenges diagnosis, staging, and treatment planning.

Both PLC and IBC lack well-defined masses, typically presenting on MRI as asymmetric density, skin thickening, or diffuse non-mass enhancement. Conventional imaging has limited utility: mammography may be poorly tolerated and fails to detect the primary lesion in over 40% of IBC cases (16), while ultrasonography poorly delineates diffuse infiltration and superficial skin involvement. MRI identifies over 90% of lesions, and PET/CT is preferred for distant metastasis detection, but access is limited in resource-constrained settings, leading to inaccurate staging. These overlapping radiologic features support the need for early biopsy in patients with rapidly progressive breast inflammation to avoid diagnostic delay and enable timely initiation of systemic therapy. Furthermore, pathologic assessment is needed to establish both the histologic features of PLC and the clinical phenotype of IBC as inadequate sampling depth or overly localized skin biopsies may lead to missed IBC diagnoses, highlighting the need for multisite biopsy and close clinicopathologic collaboration.

PLC has a much higher HER2 positivity rate [30%–80% (17)] than classic invasive lobular carcinoma [ILC; 0.6% (17)], and IBC [40% (18)] has a higher rate than non-inflammatory breast cancer [NIBC; 25% (19)]. Both PLC and IBC exhibit a high HER2 positivity rate, suggesting that abnormal activation of the HER2 pathway may be a key driving event for this overlapping phenotype and provide an important basis for targeted therapy. Therefore, PLC combined with IBC is not a simple superposition of “morphology + phenotype”, but a biologically highly malignant synergistic combination, which determines its clinical characteristics: extremely short progression-free survival, frequent drug resistance, and poor prognosis. Since the co-occurrence of PLC and IBC is rare, evidence is limited to small series, and management often follows non-special-type invasive breast carcinoma (NST) guidelines—despite biological differences impacting treatment selection. In addition, both PLC and IBC have been described as less responsive to standard cytotoxic chemotherapy than IDC; especially when these phenotypes overlap, the probability of suboptimal response may increase further. For our case, the patient presented with stage IV disease, substantial local inflammation coexisting with distant metastases that required simultaneous systemic disease control. This dual clinical burden also increased the complexity of treatment tolerance monitoring and supportive care, particularly given the need for timely escalation of systemic therapy while maintaining functional status.

For HER2-positive advanced breast cancer, anti-HER2 targeted therapy combined with chemotherapy is core. CSCO Breast Cancer Guidelines list the TPH [CLEOPATRA trial (20)] and PyroHT [PHILA trial (21)] regimens as Category 1A Level I recommendations. Nevertheless, pertuzumab is not covered by medical insurance for the treatment of advanced breast cancer in China, With respect to therapy, the patient received first-line treatment with the PyroHT regimen. The PHILA trial showed that adding pyrotinib (irreversible pan-HER tyrosine kinase inhibitor) to trastuzumab and docetaxel significantly prolonged progression-free survival (24.3 months vs. 10.4 months; HR 0.41, 95% CI 0.32–0.53; P<0.001) in treatment-naïve HER2-positive metastatic breast cancer (21), with consistent real-world efficacy and manageable toxicity (22, 23). Dual HER2 blockade (pyrotinib + trastuzumab) acts synergistically: pyrotinib sustains intracellular HER2 inhibition, while trastuzumab mediates antibody-dependent cellular cytotoxicity, improving tumor control and potentially delaying resistance (24).

Despite our patient achieving partial response after four cycles of PyroHT, progression occurred after ten cycles, consistent with the median duration of response in advanced HER2-positive IBC. The relatively short progression-free interval highlights the high aggressiveness of HER2-positive PLC/IBC overlap and early activation of acquired resistance. Therapeutic resistance remains a major limitation in HER2-positive breast cancer. Common resistance mechanisms include reduced HER2 availability (downregulated expression, truncated p95HER2 (24), decreased gene amplification), bypass signaling activation (PI3K-AKT-mTOR, EGFR/HER3, c-MET pathways (25)), and tumor microenvironment remodeling may lead to drug resistance by inducing immune escape programs (including PD-L1 upregulation) and proangiogenic signals.

The incorporation of zoledronic acid every 28 days for bone metastasis management follows current evidence-based guidelines and represents the standard of care for patients with breast cancer bone involvement (26). Recent systematic reviews and meta-analyses continue to support the efficacy of zoledronic acid in reducing skeletal-related events, as it can improve patients’ quality of life and provide survival benefits for those with metastatic breast cancer (27, 28). In addition, current international guidelines, including updated ESMO and NCCN recommendations, recommend zoledronic acid as the preferred bisphosphonate for bone metastasis management, with the standard dosing of 4 mg intravenously every 28 days as was utilized in this case (29).

Upon disease progression, T-DXd was administered as a pivotal salvage therapy for HER2-positive breast cancer after prior anti-HER2 treatment. In the DESTINY-Breast03 trial, T-DXd demonstrated superior efficacy compared with T-DM1 in previously treated HER2-positive breast cancer, including patients with brain metastases (30). As an antibody-drug conjugate, T-DXd consists of an anti-HER2 monoclonal antibody linked to a potent topoisomerase I inhibitor payload via a cleavable linker, enabling robust intracellular cytotoxicity upon internalization.

Importantly, T-DXd exerts a distinct bystander effect, which is highly advantageous for diffuse infiltrative diseases such as inflammatory breast cancer. In our case, T-DXd treatment resulted in marked tumor regression and significant improvement in breast erythema and pain. This clinical response supports the therapeutic rationale of T-DXd in HER2-positive breast cancer with an IBC-like phenotype, via both targeted cytotoxicity and bystander-mediated eradication of infiltrative tumor components.

Recently, the DESTINY-Breast04 study reported its benefits to HER2-low disease, though our case involved classical HER2-positive status (31). The marked improvement in breast symptoms and lesion reduction after four cycles of T-DXd in our patient is consistent with these trial results, supporting the drug’s ability to overcome resistance mechanisms even in aggressive histological and clinical subtypes. comprehensive genomic profiling is recommended to clarify resistance mechanisms and guide subsequent therapy. Recommended genetic testing includes ERBB2 amplification/mutations, PI3K-AKT-mTOR pathway genes (PIK3CA, AKT1, PTEN), bypass signaling genes (EGFR, MET), PLC-related genes (CDH1, TP53), and biomarkers for endocrine therapy, PARP inhibitors and immunotherapy (ESR1, BRCA1/2, PD-L1, TMB, MSI). Serial ctDNA monitoring can dynamically track clonal evolution and emergent resistance mutations. Genomic characterization helps identify resistance to anti-HER2 regimens and enables mechanism-driven salvage strategies, including novel anti-HER2 ADCs, pan-HER TKIs, PI3K/AKT/mTOR inhibitors, and immunotherapy combinations, thereby supporting individualized treatment optimization.

Common and notable T-DXd-related adverse events (AE) include infusion reactions, gastrointestinal (nausea/vomiting, constipation, diarrhea, decreased appetite), hematologic (neutropenia, febrile neutropenia, anemia, thrombocytopenia), respiratory (interstitial lung disease [ILD]/pneumonitis), cardiovascular (left ventricular ejection fraction reduction), hepatic (transaminase elevation) AE, and alopecia, fatigue. Despite its marked efficacy, close monitoring of treatment-related AEs is essential. Key toxicities (myelosuppression, gastrointestinal reactions) are mostly manageable with supportive care (32). Critically, ILD/pneumonitis is a potentially fatal complication, requires prompt symptom recognition (cough, dyspnea, hypoxia), early radiologic evaluation, and timely corticosteroid intervention (33). Close prospective monitoring ensures safety. An urgent clinical challenge is optimizing AE management to improve T-DXd tolerance, adherence, and efficacy. Enhancing AE understanding and standardizing management can improve medication experience and adherence, maximizing T-DXd’s anti-tumor effect and clinical benefits.

Surgical evaluation is a key part of the treatment decision for this case. The patient was initially diagnosed with stage IV metastatic breast cancer with lymph node and bone metastases, so surgical treatment was excluded early. Per clinical guidelines and consensus, the core goal of metastatic breast cancer treatment is to control systemic disease, relieve symptoms, and prolong survival; surgery, as a local treatment, is not routinely used for initial treatment of metastatic tumors and is only cautiously considered in specific cases. Given the biological characteristics of PLC and IBC, occult local breast infiltration may persist even with complete remission of distant metastases, and IBC’s diffuse infiltration predisposes to local recurrence. However, no clear guidelines recommend surgery for such patients after complete remission. MDT discussion concluded that surgery offers limited benefit, may increase patient trauma, impair quality of life, and not improve long-term survival; thus, surgical intervention is not planned post-complete remission of metastases.

This case also demonstrates the significance of multidisciplinary tumor board review in guiding therapy. The decisions to initiate dual HER2 blockade, later introduce T-DXd, and maintain bone-targeted therapy with zoledronic acid were all made within a structured multidisciplinary framework and in accordance with national guidelines. Such an approach is essential in rare and complex cases where evidence is limited.

## 4 Strengths and limitations

The main strength of this report is the detailed account of a rare breast cancer subtype, combining multimodal imaging, thorough pathological evaluation, and documentation of sequential HER2-targeted treatments with trastuzumab, pyrotinib, and T-DXd. The favorable response to antibody–drug conjugate therapy offers a clinical perspective on treatment sequencing for aggressive lobular carcinoma with inflammatory features. However, several limitations should be noted. First, comprehensive genomic profiling was not performed, which might have revealed additional therapeutic options. Second, following diagnosis of advanced breast cancer, the patient received 10 cycles of first-line PyroHT regimen, with a PFS of 7 months. Upon disease progression, second-line T-DXd was initiated. To date, the patient has achieved a PFS of 13 months and remains in partial response (PR), the follow-up duration was relatively short, limiting assessment of long-term outcomes. Continued long-term follow-up will be performed to regularly assess the primary breast lesion and metastatic sites, and timely adjustments to the therapeutic strategy will be made to optimize the patient’s overall survival (OS).Lastly, as a single case, its findings cannot be generalized. Despite these, our report provides clinical insight into the limited literature on the rare coexistence of PLC and the IBC phenotype.

## 5 Conclusion

This case represents the first report of HER2-positive advanced PLC with an IBC phenotype responding to both PyroHT and T-DXd. The combination of subtle lobular imaging features and diffuse inflammatory changes required histologic confirmation. Molecular profiling identified a HER2-positive, hormone receptor-negative subtype, which led to the use of HER2-targeted therapy. Sequential treatment with dual HER2 blockade and later T-DXd provided initial clinical benefit despite eventual resistance. Together, this case indicates that management of rare histologic variants should be guided by molecular features and supported by multidisciplinary decision-making.

## Data Availability

The raw data supporting the conclusions of this article will be made available by the authors, without undue reservation.
